# KAT8 beyond Acetylation: A Survey of Its Epigenetic Regulation, Genetic Variability, and Implications for Human Health

**DOI:** 10.3390/genes15050639

**Published:** 2024-05-17

**Authors:** Lindsey Yoo, David Mendoza, Allison J. Richard, Jacqueline M. Stephens

**Affiliations:** 1 Adipocyte Biology Laboratory, Pennington Biomedical, Baton Rouge, LA 70808, USA; lking51@lsu.edu (L.Y.); david.mendoza@pbrc.edu (D.M.); allison.richard@pbrc.edu (A.J.R.); 2 Department of Biological Sciences, Louisiana State University, Baton Rouge, LA 70803, USA

**Keywords:** epigenetic regulation, single nucleotide polymorphism (SNPs), multiprotein complexes, chromatin remodeling, cellular homeostasis

## Abstract

Lysine acetyltransferase 8, also known as KAT8, is an enzyme involved in epigenetic regulation, primarily recognized for its ability to modulate histone acetylation. This review presents an overview of KAT8, emphasizing its biological functions, which impact many cellular processes and range from chromatin remodeling to genetic and epigenetic regulation. In many model systems, KAT8’s acetylation of histone H4 lysine 16 (H4K16) is critical for chromatin structure modification, which influences gene expression, cell proliferation, differentiation, and apoptosis. Furthermore, this review summarizes the observed genetic variability within the *KAT8* gene, underscoring the implications of various single nucleotide polymorphisms (SNPs) that affect its functional efficacy and are linked to diverse phenotypic outcomes, ranging from metabolic traits to neurological disorders. Advanced insights into the structural biology of KAT8 reveal its interaction with multiprotein assemblies, such as the male-specific lethal (MSL) and non-specific lethal (NSL) complexes, which regulate a wide range of transcriptional activities and developmental functions. Additionally, this review focuses on KAT8’s roles in cellular homeostasis, stem cell identity, DNA damage repair, and immune response, highlighting its potential as a therapeutic target. The implications of KAT8 in health and disease, as evidenced by recent studies, affirm its importance in cellular physiology and human pathology.

## 1. Introduction

Lysine acetyltransferase 8, also known as KAT8, is widely recognized for its role in the acetylation of histones, a crucial epigenetic mark that regulates gene expression. KAT8 was initially called MOF (males-absent-on-the-first) in *Drosophila melanogaster* due to mutant flies demonstrating male-specific mortality when unable to undergo sex chromosomal dosage compensation [1]. As part of the male-specific lethal (MSL) complex, MOF enhances the transcription of genes on the single X chromosome in males by acetylating H4 at lysine 16. It also plays a role in the non-specific lethal (NSL) complex, which regulates gene expression on both X chromosomes in females [2]. The human homolog of MOF was identified as part of the MYST (MOZ, Ybf2/Sas3, Sas2, and Tip60) family of histone acetyltransferases [3]. This family of histone acetyltransferases is classified based on the presence of a highly conserved MYST domain that contains an acetyl-CoA binding site and a C2HC zinc finger motif, which are critical for the KAT8’s acetyltransferase activity [4]. It is important to note that, although early literature considered these enzymes as histone acetyltransferases (HATs), they also target proteins other than histones; therefore, they are now regarded as lysine acetyltransferases (KATs) [5].

The enzymatic action of lysine acetyltransferases (KATs) requires the transfer of acetyl groups from acetyl CoA to certain lysine residues of proteins, which are frequently histones [4]. The process of histone acetylation, which is critical in the control of chromatin architecture and the regulation of gene expression, can be reversed by histone deacetylases (HDACs), which are also more broadly referred to as lysine deacetylases (KDACs) [6]. Together, KATs and KDACs preserve a stable equilibrium of acetylation and deacetylation, which maintains optimal cellular function [5]. The most studied substrate of KAT8 is histone H4 lysine 16 (H4K16), although KAT8 can also acetylate H4K5 and H4K8 [6]. Acetylation of H4K16 can cause the chromatin structure to loosen, promoting activation of gene transcription. This process effects a variety of biological processes, including cell proliferation, differentiation, and cell death [7,8]. Additionally, atypical KAT8 expression and/or function can cause abnormalities in a variety of cell processes including the cell cycle, proliferation, DNA damage repair, early embryonic development, as well as different types of cancers [5,6,9].

Beyond histone acetylation, KAT8 and other KATs have been proposed to have other enzymatic activities, including propionylation and crotonylation, indicating a broader functional scope that is not fully understood due to current limitations in research reagents [10,11]. KAT8 also exhibits a unique versatility by targeting substrates in both the nucleus and cytosol [12]. The tumor suppressor protein p53 can be acetylated by KAT8 to regulate the cell cycle and apoptosis [13,14]. Since KAT8 can be present in the cytosol, it is likely that other non-nuclear KAT8 substrates will be identified in the cytosol. The broad functionality of KAT8 underscores its crucial role in maintaining cellular homeostasis and finely regulating gene expression. The current exploration of KAT8’s activities reveals a complex enzyme with broad biological functions, underlining the importance of continued research into its various substrates and regulatory mechanisms.

## 2. KAT8 SNPs: Genetic Variability and Implications

Advances in genomic sequencing and bioinformatics have allowed for the identification of single nucleotide polymorphisms (SNPs), revealing a spectrum of variations within the KAT8 locus, which can significantly impact *KAT8* gene function and its effects on broader regulatory networks. This could include variations that might affect KAT8’s ability to acetylate histone marks, thereby altering the expression of genes under its regulatory domain. The genetic variability in *KAT8*, evidenced by its SNPs cataloged in Ensembl “https://www.ensembl.org (accessed on 8 May 2024)”, has many implications for human diseases. In this review, we delve into the diverse phenotypic outcomes associated with SNPs in the *KAT8* gene, demonstrating the extensive impact of these genetic variations. These associations indicate that alterations in KAT8-mediated epigenetic regulation can contribute to disease pathogenesis, reflecting KAT8’s essential role in maintaining genomic stability and regulating gene expression.

Multiple SNPs associated with *KAT8* have been cataloged, each with distinct phenotypic consequences. For instance, the splice region variant rs9925964 has been extensively cited for its association with body mass index (BMI) fluctuations [15], physical activity [16], and longitudinal BMI [17]. Furthermore, the intronic SNP rs59735493 has been linked to Alzheimer’s disease [18] and anxiety [19], underscoring the gene’s broad phenotypic impact beyond metabolic traits. Another SNP, rs1549293, located in the 3′ UTR, is connected to metabolic and physical features, including waist circumference [20]. Moreover, *KAT8* SNPs have been identified in various genomic contexts, ranging from intronic and intergenic regions to splice sites and noncoding transcript exons, indicating the gene’s complex regulatory network. For example, rs138259061, an intron variant, is associated with triglyceride levels [21], while rs11865499, a noncoding transcript exon, affects body height [22]. Additional SNPs associated with *KAT8*, along with their respective phenotypic impacts are shown in Table 1 and Appendix A, Table A1, Table A2, Table A3, Table A4, Table A5, Table A6 and Table A7.

Li–Ghorbani–Weisz-Hubshman syndrome, marked by the missense variant rs748699921 in the coding region of the *KAT8* gene, is a rare genetic disorder characterized by its profound impact on cerebral development and the manifestation of syndromic intellectual disability [23]. The syndrome is distinguished by a spectrum of neurological and developmental challenges, including significant delays in reaching developmental milestones, intellectual disability ranging from mild to severe, and potential behavioral and emotional difficulties. Neurological symptoms may encompass seizures, issues with muscle tone, and coordination problems. Additionally, individuals with this condition can exhibit unique facial dysmorphisms and various physical anomalies, which are critical for diagnosis. This disease underscores the essential role of the KAT8 for proper neural development, as it ensures the expression of genes critical for neuronal differentiation and function. Disruptions in KAT8’s activity can lead to impaired chromatin remodeling and gene expression, resulting in significant neurological deficits, demonstrating the gene’s indispensable role in maintaining brain health. [23].

The identification of these SNPs has been facilitated by advances in genomic technologies and bioinformatics, enabling a more nuanced understanding of the genetic determinants of health and disease. As research progresses, the catalog of *KAT8*-associated SNPs continues to expand, offering new insights into the gene’s role in human physiology and pathology. This growing body of evidence emphasizes the importance of KAT8 as a key player in epigenetic regulation and its potential as a target for therapeutic intervention in various conditions. Integrating these findings with broader KAT8 research can provide comprehensive insights into the gene’s multifaceted roles.

## 3. Structural Insights into KAT8 and Its Complexes

**Figure 1:**
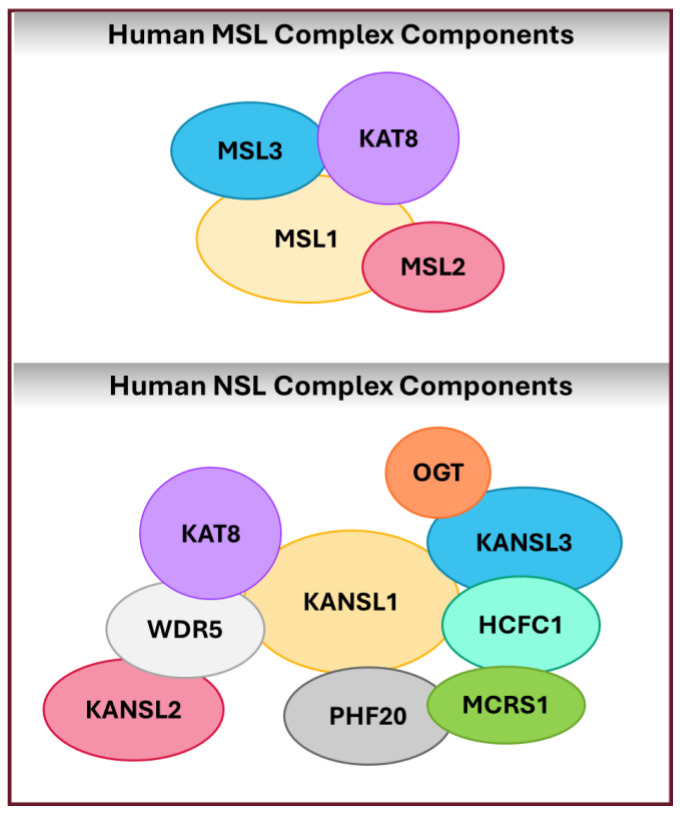
Schematic representation of the two major human protein complexes involving KAT8. Top: The male-specific lethal (MSL) complex components, showing KAT8’s interaction with MSL1, MSL2, and MSL3. Bottom: The non-specific lethal (NSL) complex component showing KAT8’s interaction with other components, including KANSL1, KANSL2, KANSL3, PHF20, HCF1, WDR5, OGT, and MCRS1.

KAT8 plays a critical role in epigenetic regulation through its involvement with two key multiprotein complexes: the male-specific lethal (MSL) and non-specific lethal (NSL) complexes. While some components of these complexes are conserved between *D. melanogaster* and humans, the specific roles of these components in humans are poorly understood. In the *D. melanogaster* MSL complex, MSL1 (MSL complex subunit 1) serves as a scaffold facilitating the assembly of additional components [24]. MSL2 (MSL complex subunit 2) is vital for the complex’s stabilization and regulation, leveraging its E3 ubiquitin ligase activity to maintain the proper stoichiometry and functionality of the complex components [25], whereas the MSL3 (MSL complex subunit 3) targets the complex to chromatin through its chromodomain, which recognizes methylated histone peptides [26]. The assembly and targeting of the complex are intricately regulated by non-coding RNAs (ncRNAs) and the RNA helicase MLE (maleless) protein, guiding the complex to specific genomic sites for targeted epigenetic regulation [27]. KAT8’s primary role in the MSL complex appears to be highly specific, as it uses its HAT activity to add an acetyl group specifically to lysine 16 on histone H4 (H4K16) [28]. In humans, a homologous MSL complex consisting of KAT8, MSL1, MSL2, and MSL3 (Figure 1) has been identified and is responsible for the majority of H4K16ac across all chromosomes [28,29]. This activity is essential for transcription regulation and cell cycle progression, highlighting a divergent evolutionary path for MSL complex functions in the absence of direct analogs to *D. melanogaster*’s dosage compensation regulators, such as the MLE protein, and the non-coding RNAs on the X (roX) [27,30] The conservation of core components in the human MSL complex emphasizes its significance in mammalian epigenetic regulation and warrants further exploration to understand these complex components and their interactions in humans.

Orthologs corresponding to the NSL complex have been discovered across a diverse spectrum of species, indicating its widespread conservation [29]. Due to the complex’s potential role in various diseases, which will be detailed in later sections, the human NSL complex has been more extensively studied even though it was discovered later than the MSL complex. At its core, the human NSL complex comprises the unique members of KAT8, KANSL1 (KAT8 regulatory NSL complex subunit 1), KANSL2, KANSL3, and PHF20 (PHD finger protein 20 (Figure 1). Additional components, shared with other chromatin-modifying complexes, include MCRS1 (microspherule protein 1), WDR5 (WD repeat domain 5), OGT (O-Linked N-acetylglucosamine transferase 1), and HCF1 (host cell factor 1) [31]. Within this complex, KAT8 still possesses HAT activity, but here it can acetylate histone H4 at multiple lysine residues, beyond H4K16, such as H4 at lysines 5 and 8 (H4K5ac and H4K8ac) [28]. KANSL1 and KANSL2 act as scaffolding proteins, ensuring complex stability and interacting with WDR5 for chromatin recruitment [31]. KANSL3, beyond its own scaffolding role, might play a part in mitotic spindle assembly [32]. PHF20, through its PHD finger, specifically recognizes H3K4me2 marks, directing the NSL complex to specific genes for transcriptional regulation [33]. The specific makeup and activity of these complexes lead to varied functional consequences in different biological contexts, emphasizing the complexity and significance of KAT8 and its roles in cellular processes.

## 4. KAT8 in Cellular Homeostasis and Transcriptional Regulation

KAT8, whether part of the NSL or MSL complex, plays a pivotal role in regulating transcriptional activity. Although it can potentially acetylate non-histone substrates, most of the current literature demonstrates that KAT8 regulates transcription through its histone acetyltransferase activity and its interaction within these complexes. KAT8 significantly impacts cellular homeostasis by regulating DNA accessibility and transcription factor recruitment to control gene expression in response to various signals [31]. This regulation plays a role in cellular homeostasis, cell differentiation [5], tissue development [23,34], and stress responses [35]. Important for both cellular and organismal health, the NSL complex is a vital regulator of housekeeping genes, notably in *D. melanogaster*, by promoting RNA Polymerase II binding to gene promoters [36], which is required for efficient transcription of genes integral to cellular functions in various tissues. The NSL complex’s role extends beyond housekeeping gene regulation to transcriptional control of a wide array of genes [31]. The NSL complex also stabilizes the nuclear envelope through the acetylation of lamin A/C by KAT8, preventing nuclear blebbing and micronuclei formation, and thus maintaining nuclear integrity [37]. Loss of KAT8 function results in deacetylation of lamin A/C, leading to nuclear instability and significant genomic alterations, emphasizing its critical structural role alongside its transcriptional regulation [37].

The NSL complex promotes the recruitment of Bromodomain-Containing Protein 4 (BRD4), a member of the BET (Bromodomain and Extra-Terminal domain) family of proteins, which plays a significant role in the elongation phase of transcription by recognizing acetylation marks on histones and facilitating the formation of a functional elongation complex [28]. This action is crucial for recruiting transcriptional machinery to chromatin to support gene expression that is critical for cell cycle, development, and stress responses [38]. The interaction between the NSL complex and BRD4 is vital for maintaining cellular balance, and disruptions are linked to serious health conditions, such as Koolen–de Vries syndrome (KdVS) [39]. KdVS, resulting from mutations in the *KANSL1* gene, is characterized by developmental delay, intellectual disability, and distinctive facial features [40]. Through its collaborative role with BRD4, the NSL complex’s acetylation activities regulate a variety of cellular functions and can impact development.

The NSL complex collaborates with chromatin modifiers, like the Mixed Lineage Leukemia (MLL/SET domain) complexes, to enhance histone H3 lysine 4 di-methylation (H3K4me2) through histone acetylation [41] and promote transcriptional activation [42]. The MLL/SET complex works with the NSL complex to maintain active chromatin states that are essential for development, differentiation, and cellular responses [31]. This partnership highlights how acetylation by the NSL complex and methylation by MLL/SET are interconnected, ensuring appropriate gene expression. The NSL complex’s ability to target multiple H4 lysines for acetylation broadens its regulatory scope, impacting essential genes by promoting H3K4me2 in an acetylation-dependent manner [41]. In essence, the NSL complex, which includes KAT8, possesses the ability to modulate transcription through various mechanisms. It achieves this by facilitating histone marks linked to chromatin remodeling and accessibility, as well as recruiting the necessary transcriptional machinery for initiation and/or elongation.

The MSL complex, like NSL, involves KAT8’s acetyltransferase activity, particularly targeting histone H4 at lysine 16 (H4K16ac), to modify chromatin structure and facilitate transcriptional activation [24,25]. This acetylation is crucial for counteracting chromatin compaction, thereby enhancing the accessibility of the DNA to transcription machinery. Unlike in *D. melanogaster*, where the MSL complex is directly involved in dosage compensation, it does not perform this function in humans. Instead, the human MSL complex plays a critical role in the global regulation of gene expression across all chromosomes [27,29]. The MSL complex primarily targets H4K16ac, while the NSL complex has a broader substrate specificity, catalyzing H4K5ac and H4K8ac in addition to H4K16ac. This broader activity pattern enables the NSL complex to regulate transcription initiation more directly at transcription start sites (TSSs) through H4K5 and H4K8 acetylation [28].

Despite these differences, both complexes have essential roles in regulating chromatin dynamics and maintaining proper gene expression. The MSL complex directly influences global chromatin accessibility through its specific modification of H4K16, while the NSL complex is crucial for transcription initiation and cell proliferation [28]. The synergistic action of the MSL and NSL complexes underscores the versatile role of KAT8 in regulating key aspects of chromatin dynamics and transcriptional activity, impacting various fundamental cellular processes, and contributing to organismal health and disease [31]. Specifically, the depletion of either complex can lead to significant transcriptional dysregulation, revealing the importance of KAT8-associated acetyltransferase activity in coordinating cellular homeostasis [28].

## 5. KAT8’s Role in Stem Cell Identity and Differentiation

Beyond its recognized molecular functions in chromatin modification and transcriptional regulation, KAT8 is also involved in maintaining stem cell identity [43] and modulating the differentiation of various cell types [5]. In mouse embryonic stem cells (mESCs), KAT8 is crucial for pluripotency and early development [44]. Both NSL and MSL complexes modulate transcription in mESCs by regulating specific and overlapping sets of genes, with NSL predominantly associating with promoters, and MSL with gene bodies [44]. Notably, the NSL complex regulates cellular proliferation and maintains cellular homeostasis by acetylating histones to enhance chromatin accessibility, thereby facilitating the transcription of genes that govern cell growth, division, and survival [28]. This action ensures that cells can efficiently respond to growth signals and maintain essential functions under varying physiological conditions. By contrast, the MSL complex in mESCs plays a critical role in silencing a subset of genes while ensuring others are prepared for activation during differentiation [44]. There is some evidence that the MSL complex participates in the X-chromosome inactivation during differentiation of female mouse embryonic stem cells by attaching itself to the Tsix/Xist locus [43].

KAT8’s role in cellular differentiation notably extends to adipogenesis, a process critical for the development of fat cells. KAT8 was found to be required for the in vitro differentiation of 3T3-L1 preadipocytes into mature adipocytes [5]. The loss of KAT8 expression blocked the ability of preadipocytes to accumulate lipid and induce the expression of key adipocyte markers [5]. Interestingly, the necessity of KAT8 is specifically associated with modulating the mitotic clonal expansion phase of adipocyte differentiation, as knockdown of its expression post-mitotic clonal expansion or in fully differentiated adipocytes does not affect lipid accumulation or the expression of adipocyte marker genes [5]. This specific involvement of KAT8 underlines its significant yet nuanced role in cellular differentiation. However, contrary to previous reports where STAT5B (Signal transducer and activator of transcription 5B) was identified as a negative regulator of adipogenesis by modulating KAT8 expression [45], these findings suggest a different interaction where KAT8 actually plays a positive role in the early stages of adipocyte differentiation. This discrepancy calls for further research to fully understand the mechanisms of KAT8 in adipogenesis and its potential *in vivo* effects, which could provide foundational insights into cellular differentiation processes and pave the way for future studies on metabolic regulation.

KAT8’s impact on cellular differentiation extends to a variety of other cell types. Studies have suggested KAT8’s involvement in osteoblast differentiation, where it promotes the transcriptional activation of osteogenic markers, such as Runx2 and Osterix, which are essential for bone health [46]. KAT8 also plays a pivotal role in T-cell maturation, where its deletion leads to severe defects in T-cell receptor rearrangement and a consequential increase in genomic instability [47]. The NSL complex, particularly through its components KANSL2 and KANSL3, is essential for the transcriptional regulation of intraciliary transport genes that influence cilia assembly and microtubule dynamics [48]. This regulatory activity is crucial for cellular differentiation, which impacts kidney health, by maintaining podocyte function, as the deletion of *KANSL2* or *KANSL3* in podocytes leads to severe kidney dysfunction [48]. Furthermore, studies show that KAT8 influences acute myeloid leukemia (AML) differentiation by suppressing *MN1* (meningioma 1) expression, a gene associated with rapid leukemia onset when overexpressed [49]. These multifaceted roles of KAT8 across different cell types illustrate its critical influence on stem cells and cellular differentiation, emphasizing its essential contribution to the development and specialized function of various tissues.

## 6. KAT8 in DNA Damage Response and Repair

KAT8’s capacity to acetylate H4K16 is linked to its regulation of both autophagy and the DNA damage response [50]. In MEFs, as well as in various human cancer cell lines, the induction of autophagy leads to decreased H4K16 acetylation, which regulates genes that are critical for managing cellular stress and DNA repair [50]. A feedback loop exists, where changes in H4K16 acetylation during autophagy impacts gene expression in MEFs, crucial for the cellular decision between survival and death under stress, thus affecting DNA repair mechanisms [50]. Furthermore, treatment with rapamycin, a known inducer of autophagy, leads to reduced H4K16 acetylation. Interestingly, this autophagic response depends on the activity of KAT8, but not its deacetylase counterpart SIRT1 [50]. Additionally, KAT8’s role in acetylating H4K16 is vital for DNA damage response and repair across multiple human cell models (HEK293 and HL60 cells), as its reduction is associated with impaired response to DNA double-strand breaks (DSBs), specifically affecting both non-homologous end joining (NHEJ) and homologous recombination repair pathways in HEK293 cells [51]. Notably, KAT8 physically interacts with DNA-dependent protein kinase catalytic subunit (DNA-PKcs), a key enzyme in the NHEJ pathway that is essential for effective DNA repair by direct rejoining of DNA ends. In KAT8-deficient cells exposed to ionizing radiation, reduced DSB repair is associated with decreased ATM (ataxia telangiectasia mutated)-dependent phospho-activation of DNA-PKcs [51]. Thus, the impairment of DNA repair responses due to loss of KAT8 activity may stem from multiple mechanisms, such as deficient H4K16 acetylation, as well as defective recruitment or activation of repairosome proteins.

## 7. KAT8 in Mitochondrial Function

KAT8 also plays a role in modulating mitochondrial function through its ability to acetylate non-histone substrates. A loss of *KAT8* in cardiomyocytes results in a down regulation of mitochondrial metabolism and oxidative phosphorylation [52]. In this study, the authors demonstrate a pool of KAT8 in mitochondria that regulates transcription of mitochondrial DNA [52]. Additionally, KAT8 can acetylate COX17, which is a complex IV assembly factor in mitochondria, and depletion of COX17 or expression of unacetylated forms of the protein leads to defects in mitochondrial structure and function in mouse embryonic fibroblasts [53]. Moreover, fibroblasts from patients with MOF syndrome (a disorder caused by mutations in the *KAT8* gene) have shown respiratory defects that could be restored by mitochondrially targeted MOF [53]. In addition to mitochondrial structure and function, KAT8 also plays a role in the selective degradation of damaged or dysfunctional mitochondria by autophagy. This is seen in neuroblastoma cells where the dual inhibition of KAT5 and KAT8 inhibits the initial steps of PTEN-induced kinase 1 (PINK1)-dependent mitophagy [54]. These findings show connections between KAT8 and mitochondrial function through protein acetylation, regulation of mitochondrial dynamics, and modulation of mitophagy. Yet, the specific targets and pathways through which KAT8 acts remain largely unknown. Also, the similarities and differences in the mechanisms by which KAT8 impacts mitochondrial function in different cell types remains to be elucidated. Since KAT8 function varies in a manner dependent on cell type, developmental stage, and environmental or physiological conditions, it will be important to consider these complexities when examining the roles of KAT8 in mitochondria regulation.

## 8. KAT8 in Inflammation and Immune Response

Recent research has focused on the significance of epigenetic modulators, including KAT8, in regulating the activation pathways of macrophages [55,56]. Macrophages are crucial for the immune system and are widely distributed across both lymphoid and non-lymphoid tissues [57]. They have many important roles, ranging from initiating the immune response to pathogens and managing inflammatory processes to facilitating wound healing and tissue remodeling, such that macrophage dysfunction can contribute to the pathogenesis of non-healing wounds [58,59]. A myeloid-specific *KAT8*-knockout model has altered inflammatory cytokine production in macrophages during the inflammatory phase of wound repair. Moreover, the overexpression of *KAT8* in wound macrophages can drive the expression of inflammatory cytokines through the acetylation of histone H4K16 [60]. The production of cytokines by macrophages can initiate a cascade of inflammatory mediators, potentially leading to extensive tissue damage [59]. The precise mechanisms through which KAT8 influences macrophage function and its role in acetylating histones to modulate macrophage gene expression and function are largely unexplored.

Since KAT8 can affect macrophage function and inflammatory cytokine production, modulating KAT8 function could potentially be a therapeutic target for chronic inflammatory diseases. Increased KAT activity and reduced KDAC activity have been observed in genetically modified asthmatic mice [61]. Notably, treatment with the KAT8 inhibitor, MG149, decreased pro-inflammatory gene expression in the murine lungs [61,62]. Increased histone acetylation in the tumor necrosis factor (*Tnfa*) and monocyte chemotactic protein 1 (*Mcp1*/*Ccl2*) inflammatory cytokine genes has also been associated with fatty liver disease in mouse models of obesity [63]. Furthermore, there is an association of fibrosis with KAT8. Loss of KAT8 significantly inhibits profibrogenic gene expression, including *Acta2* (alpha actin 2) and *Col1a1* (collagen type 1 alpha 1), through the decreased expression of *Ncf1* (neutrophil cytosolic factor 1) and *Ncf2* in primary mouse LX-2 hepatic stellate cells (HSCs) [64].

The role of KAT8 in the stress response within immune cells reveals a connection between epigenetic regulation and immune function. Through histone acetylation, KAT8 works as a critical regulator of immune cell behavior under stress conditions, such as viral infections. However, KAT8 can also influence macrophage function in response to viral infections by directly acetylating a non-histone protein, interferon regulatory factor 3 (IRF3) [65]. KAT8’s expression in immune cells, specifically macrophages and dendritic cells, is crucial for its role in selectively inhibiting IFN-I production in response to RNA and DNA virus challenges. The acetylation of IRF3 by KAT8 impedes IRF3’s recruitment to IFN-I gene promoters, downregulating its transcriptional activity and reducing the antiviral innate immune response [60,65]. This is one example of how KAT8 can modulate the antiviral response and effects of cellular stress in diseases characterized by altered immune states.

Future studies could delve deeper into several aspects of our understanding of KAT8’s role in inflammation and immune response. Firstly, investigating the specific molecular mechanisms underlying KAT8-mediated regulation of inflammatory cytokine production in macrophages, including the identification of downstream targets and signaling pathways affected by KAT8 activity, would provide valuable insights. Additionally, exploring the interplay between KAT8 and other epigenetic modifiers or transcription factors known to regulate immune gene expression could elucidate complex regulatory networks governing immune responses. Furthermore, examining the impact of KAT8 dysregulation in various disease contexts, such as infectious diseases, autoimmune disorders, and cancer, could uncover potential therapeutic strategies targeting KAT8 for immune modulation. Lastly, elucidating the role of KAT8-mediated histone acetylation versus non-histone protein acetylation in immune cell function and inflammation, as well as other cellular functions would contribute to comprehensive understanding of KAT8’s diverse biological functions and involvement in disease pathogenesis.

## 9. Conclusions

**Figure 2:**
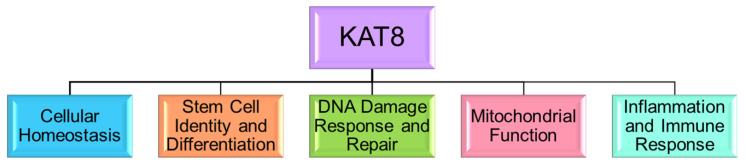
Cellular roles of KAT8.

KAT8 functions beyond its well-known involvement in histone acetylation and chromatin remodeling by also modulating acetylation and, potentially, other post-translation modifications of non-histone substrates. As summarized in Figure 2, KAT8 is essential for basic cellular processes, such as homeostasis, proliferation, and differentiation, as well as more intricate functions, like DNA repair, mitochondrial dynamics, and immune responses.

The versatility of KAT8, demonstrated by its interactions with both MSL and NSL complexes, underscores its adaptability and critical role in various cellular environments. Additionally, the genetic diversity within the *KAT8* gene, particularly with respect to SNPs, provides insights into its distinct impacts on human health and disease phenotypes, suggesting potential avenues for therapeutic development. Ongoing research into KAT8 is needed to reveal further details about its regulatory actions and partnerships, potentially leading to innovative treatments for conditions influenced by epigenetic imbalances. KAT8 serves as a prime example of the intricate nature of epigenetic regulation and its substantial influence on biological systems, affirming its importance in both physiology and disease.

## Figures and Tables

**Table 1:** *KAT8* Variant types and phenotypic implications.

SNP	Variant Type	Phenotypic Consequence(s)	Total Number of References
*rs9925964*	Splice Region	Body mass index, longitudinal BMI measurement, physical activity measurement	27
*rs59735493*	Intron	Alzheimer’s disease, anxiety	19
*rs749767*	Intergenic (*BCKDK*, *KAT8*)	central corneal thickness, corneal resistance factor	8
*rs1549293*	3 Prime UTR	Body mass index, longitudinal BMI measurement, physical activity measurement, waist circumference, triglyceride measurement	7
*rs138259061*	Intron	Triglyceride levels	3
*rs11865499*	Noncoding Transcript Exon	Body height	2
*rs61320757*	Intron	Brain measurement—vertex-wise sulcal depth	1
*rs368991827*	Intron	Prostasin levels	1
*rs748699921, 6+*	Missense	Li–Ghorbani–Weisz-Hubshman syndrome	1

The table displays the catalog names of single nucleotide polymorphisms (SNPs) within the *KAT8* gene, including their variant types—such as splice region, intron, intergenic, 3′ UTR, and noncoding transcript exon—and corresponding phenotypic outcomes. Total number of references for each association are shown on the righthand side of the table. The exact references for each SNP are shown in Table A1, Table A2, Table A3, Table A4, Table A5, Table A6 and Table A7. Information was acquired via the Ensembl “https://www.ensembl.org/ (accessed on 8 May 2024)” database.

## Data Availability

The original contributions presented in this review are included in the article/Appendix A; further inquiries can be directed to the corresponding author.

## Appendix A

**Table A1:** Citation list for rs9925964.

Year	Title	Author(s)	Reference
2023	The Genetic Basis of Childhood Obesity: A Systematic Review.	Vourdoumpa A, Paltoglou G, Charmandari E.	[66]
2022	Mendelian Randomization Analysis Reveals No Causal Relationship Between Nonalcoholic Fatty Liver Disease and Severe COVID-19.	Li J, Tian A, Zhu H, Chen L, et al.	[15]
2022	Simulated distributions from negative experiments highlight the importance of the body mass index distribution in explaining depression-body mass index genetic risk score interactions.	Casanova F, O’Loughlin J, Lewis C, Frayling TM, et al.	[67]
2021	Obesity Genes and Weight Loss During Lifestyle Intervention in Children With Obesity.	Heitkamp M, Siegrist M, Molnos S, Brandmaier S, et al.	[68]
2020	Genetic risk of obesity as a modifier of associations between neighbourhood environment and body mass index: an observational study of 335 046 UK Biobank participants.	Mason KE, Palla L, Pearce N, Phelan J, et al.	[69]
2019	Bidirectional Mendelian randomization to explore the causal relationships between body mass index and polycystic ovary syndrome.	Brower MA, Hai Y, Jones MR, Guo X, et al.	[70]
2019	Height and Body Mass Index as Modifiers of Breast Cancer Risk in BRCA1/2 Mutation Carriers: A Mendelian Randomization Study.	Qian F, Wang S, Mitchell J, McGuffog L, et al.	[71]
2018	Computational analyses of obesity associated loci generated by genome-wide association studies.	Cheng M, Mei B, Zhou Q, Zhang M, et al.	[72]
2018	Associations of adult genetic risk scores for adiposity with childhood abdominal, liver and pericardial fat assessed by magnetic resonance imaging.	Monnereau C, Santos S, van der Lugt A, Jaddoe VWV, et al.	[73]
2018	A systematic analysis highlights multiple long non-coding RNAs associated with cardiometabolic disorders.	Ghanbari M, Peters MJ, de Vries PS, Boer CG, et al.	[74]
2018	Assessing the causal role of body mass index on cardiovascular health in young adults: Mendelian randomization and recall-by-genotype analyses.	Wade KH, Chiesa ST, Hughes AD, Chaturvedi N, et al.	[75]
2018	A Large Multiethnic Genome-Wide Association Study of Adult Body Mass Index Identifies Novel Loci.	Hoffmann TJ, Choquet H, Yin J, Banda Y, et al.	[76]
2018	A high throughput, functional screen of human Body Mass Index GWAS loci using tissue-specific RNAi Drosophila melanogaster crosses.	Baranski TJ, Kraja AT, Fink JL, Feitosa M, et al.	[77]
2017	Genome-wide meta-analysis of 241,258 adults accounting for smoking behaviour identifies novel loci for obesity traits.	Justice AE, Winkler TW, Feitosa MF, Graff M, et al.	[78]
2017	Gene-obesogenic environment interactions in the UK Biobank study.	Tyrrell J, Wood AR, Ames RM, Yaghootkar H, et al.	[79]
2017	Association of Body Mass Index With Cardiometabolic Disease in the UK Biobank: A Mendelian Randomization Study.	Lyall DM, Celis-Morales C, Ward J, Iliodromiti S, et al.	[80]
2016	Body mass index and psychiatric disorders: a Mendelian randomization study.	Hartwig FP, Bowden J, Loret de Mola C, Tovo-Rodrigues L, et al.	[81]
2016	Genetic Evidence for a Link Between Favorable Adiposity and Lower Risk of Type 2 Diabetes, Hypertension, and Heart Disease.	Yaghootkar H, Lotta LA, Tyrrell J, Smit RA, et al.	[82]
2016	Genetics of Obesity.	Srivastava A, Srivastava N, Mittal B.	[83]
2016	Height, body mass index, and socioeconomic status: mendelian randomisation study in UK Biobank.	Tyrrell J, Jones SE, Beaumont R, Astley CM, et al.	[84]
2016	Associations of genetic risk scores based on adult adiposity pathways with childhood growth and adiposity measures.	Monnereau C, Vogelezang S, Kruithof CJ, Jaddoe VW, et al.	[85]
2016	Genome-Wide Association Analyses in 128,266 Individuals Identifies New Morningness and Sleep Duration Loci.	Jones SE, Tyrrell J, Wood AR, Beaumont RN, et al.	[86]
2016	Obesity and Multiple Sclerosis: A Mendelian Randomization Study.	Mokry LE, Ross S, Timpson NJ, Sawcer S, et al.	[87]
2015	Physical activity, smoking, and genetic predisposition to obesity in people from Pakistan: the PROMIS study.	Ahmad S, Zhao W, Renstrom F, Rasheed A, et al.	[16]
2015	The Influence of Age and Sex on Genetic Associations with Adult Body Size and Shape: A Large-Scale Genome-Wide Interaction Study.	Winkler TW, Justice AE, Graff M, Barata L, et al.	[88]
2015	Genetic studies of body mass index yield new insights for obesity biology.	Locke AE, Kahali B, Berndt SI, Justice AE, et al.	[17]
2015	Obesity genetics in mouse and human: back and forth, and back again.	Yazdi FT, Clee SM, Meyre D.	[89]

**Table A2:** Citation list for rs59735493.

Year	Title	Author(s)	Reference
2023	Genome-wide association studies reveal shared genetic haplotypes of autoimmune rheumatic and endocrine diseases with psychiatric disorders.	Voskarides K, Giannopoulou N, Eid R, Parperis K, et al.	[90]
2023	Brain structure and allelic associations in Alzheimer’s disease.	Moon SW, Zhao L, Matloff W, Hobel S, et al.	[91]
2022	Cell type-specific histone acetylation profiling of Alzheimer’s disease subjects and integration with genetics.	Ramamurthy E, Welch G, Cheng J, Yuan Y, et al.	[18]
2022	The genetic architecture of Alzheimer disease risk in the Ohio and Indiana Amish.	Osterman MD, Song YE, Adams LD, Laux RA, et al.	[92]
2022	Identifying genetic markers enriched by brain imaging endophenotypes in Alzheimer’s disease.	Kim M, Wu R, Yao X, Saykin AJ, et al.	[93]
2022	Genome-wide association of polygenic risk extremes for Alzheimer’s disease in the UK Biobank.	Gouveia C, Gibbons E, Dehghani N, Eapen J, et al.	[94]
2022	Mining High-Level Imaging Genetic Associations via Clustering AD Candidate Variants with Similar Brain Association Patterns.	Wu R, Bao J, Kim M, Saykin AJ, et al.	[95]
2021	Genetic Variability in Molecular Pathways Implicated in Alzheimer’s Disease: A Comprehensive Review.	Vogrinc D, Goricar K, Dolzan V.	[96]
2021	A transcriptome-wide association study of Alzheimer’s disease using prediction models of relevant tissues identifies novel candidate susceptibility genes.	Sun Y, Zhu J, Zhou D, Canchi S, et al.	[97]
2021	A transcriptome-wide association study identifies novel blood-based gene biomarker candidates for Alzheimer’s disease risk.	Sun Y, Zhou D, Rahman MR, Zhu J, et al.	[98]
2021	Mapping the proteo-genomic convergence of human diseases.	Pietzner M, Wheeler E, Carrasco-Zanini J, Cortes A, et al.	[99]
2020	Identification of Novel Alzheimer’s Disease Loci Using Sex-Specific Family-Based Association Analysis of Whole-Genome Sequence Data.	Prokopenko D, Hecker J, Kirchner R, Chapman BA, et al.	[100]
2020	Interpretation of risk loci from genome-wide association studies of Alzheimer’s disease.	Andrews SJ, Fulton-Howard B, Goate A.	[101]
2020	Polygenic mediation analysis of Alzheimer’s disease implicated intermediate amyloid imaging phenotypes.	Yingxuan E, Yao X, Liu K, Risacher SL, et al.	[102]
2020	The association of clinical phenotypes to known AD/FTD genetic risk loci and their inter-relationship.	Li QS, Tian C, 23andMe Research Team, Hinds D, et al.	[103]
2020	Genomic mechanisms in Alzheimer’s disease.	Bertram L, Tanzi RE.	[104]
2019	Evaluation of the Common Molecular Basis in Alzheimer’s and Parkinson’s Diseases.	Rana P, Franco EF, Rao Y, Syed K, et al.	[105]
2019	Genome-wide meta-analysis identifies new loci and functional pathways influencing Alzheimer’s disease risk.	Jansen IE, Savage JE, Watanabe K, Bryois J, et al.	[106]
2018	Meta-analysis of genome-wide association studies for neuroticism in 449,484 individuals identifies novel genetic loci and pathways.	Nagel M, Jansen PR, Stringer S, Watanabe K, et al.	[19]

**Table A3:** Citation list for rs749767.

Year	Title	Author(s)	Reference
2022	Association of Novel Loci With Keratoconus Susceptibility in a Multitrait Genome-Wide Association Study of the UK Biobank Database and Canadian Longitudinal Study on Aging.	He W, Han X, Ong JS, Hewitt AW, et al.	[107]
2020	Genome-Wide Association Study of VKORC1 and CYP2C9 on acenocoumarol dose, stroke recurrence and intracranial haemorrhage in Spain.	Cullell N, Carrera C, Muino E, Torres-Aguila NP, et al.	[108]
2018	Pharmacogenetic studies with oral anticoagulants. Genome-wide association studies in vitamin K antagonist and direct oral anticoagulants.	Cullell N, Carrera C, Muino E, Torres N, et al.	[109]
2017	Penetrance of Polygenic Obesity Susceptibility Loci across the Body Mass Index Distribution.	Abadi A, Alyass A, Robiou du Pont S, Bolker B, et al.	[110]
2017	Cohort-specific imputation of gene expression improves prediction of warfarin dose for African Americans.	Gottlieb A, Daneshjou R, DeGorter M, Bourgeois S, et al.	[111]
2016	Longitudinal relationships between glycemic status and body mass index in a multiethnic study: evidence from observational and genetic epidemiology.	Ishola AF, Gerstein HC, Engert JC, Mohan V, et al.	[112]
2013	Genomics of ADME gene expression: mapping expression quantitative trait loci relevant for absorption, distribution, metabolism and excretion of drugs in human liver.	Schroder A, Klein K, Winter S, Schwab M, et al.	[113]
2012	Liver expression quantitative trait loci: a foundation for pharmacogenomic research.	Glubb DM, Dholakia N, Innocenti F.	[114]

**Table A4:** Citation list for rs1549293.

Year	Title	Author(s)	Reference
2022	Mendelian Randomization Analysis Reveals No Causal Relationship Between Nonalcoholic Fatty Liver Disease and Severe COVID-19.	Li J, Tian A, Zhu H, Chen L, et al.	[15]
2022	Genomics and phenomics of body mass index reveals a complex disease network.	Huang J, Huffman JE, Huang Y, Do Valle I, et al.	[115]
2022	Pleiotropic genetic architecture and novel loci for C-reactive protein levels.	Koskeridis F, Evangelou E, Said S, Boyle JJ, et al.	[21]
2019	Mapping Genome Variants Sheds Light on Genetic and Phenotypic Differentiation in Chinese.	Guo L, Ye K.	[116]
2019	Whole Genome Analyses of Chinese Population and De Novo Assembly of A Northern Han Genome.	Du Z, Ma L, Qu H, Chen W, et al.	[20]
2015	New genetic loci link adipose and insulin biology to body fat distribution.	Shungin D, Winkler TW, Croteau-Chonka DC, Ferreira T, et al.	[117]
2008	Prostasin: a possible candidate gene for human hypertension.	Zhu H, Guo D, Li K, Yan W, et al.	[118]

**Table A5:** Citation list for rs138259061.

Year	Title	Author(s)	Reference
2021	A cross-population atlas of genetic associations for 220 human phenotypes.	Sakaue S, Kanai M, Tanigawa Y, Karjalainen J, et al.	[119]
2020	Lung Development Genes and Adult Lung Function.	Portas L, Pereira M, Shaheen SO, Wyss AB, et al.	[120]
2020	Polygenic Hyperlipidemias and Coronary Artery Disease Risk.	Ripatti P, Ramo JT, Mars NJ, Fu Y, et al.	[121]

**Table A6:** Citation list for rs11865499.

Year	Title	Author(s)	Reference
2022	A saturated map of common genetic variants associated with human height.	Yengo L, Vedantam S, Marouli E, Sidorenko J, et al.	[22]
2016	Association of Forced Vital Capacity with the Developmental Gene NCOR2.	Minelli C, Dean CH, Hind M, Alves AC, et al.	[122]

**Table A7:** Citations for rs61320757, rs368991827, and rs748699921.

Year	Title	Author(s)	Reference
**rs61320757**			
2021	The genetic architecture of human cortical folding.	van der Meer D, Kaufmann T, Shadrin AA, Makowski C, et al.	[123]
**rs368991827**			
2021	Mapping the proteo-genomic convergence of human diseases.	Pietzner M, Wheeler E, Carrasco-Zanini J, Cortes A, et al.	[99]
**rs748699921**			
2020	Lysine acetyltransferase 8 is involved in cerebral development and syndromic intellectual disability.	Li L, Ghorbani M, Weisz-Hubshman M, Rousseau J, et al.	[23]
